# Two-part permutation tests for DNA methylation and microarray data

**DOI:** 10.1186/1471-2105-6-35

**Published:** 2005-02-22

**Authors:** Markus Neuhäuser, Tanja Boes, Karl-Heinz Jöckel

**Affiliations:** 1 Institute for Medical Informatics, Biometry and Epidemiology, University of Duisburg-Essen, Hufelandstr. 55, D-45122 Essen, Germany

## Abstract

**Background:**

One important application of microarray experiments is to identify differentially expressed genes. Often, small and negative expression levels were clipped-off to be equal to an arbitrarily chosen cutoff value before a statistical test is carried out. Then, there are two types of data: truncated values and original observations. The truncated values are not just another point on the continuum of possible values and, therefore, it is appropriate to combine two statistical tests in a two-part model rather than using standard statistical methods. A similar situation occurs when DNA methylation data are investigated. In that case, there are null values (undetectable methylation) and observed positive values. For these data, we propose a two-part permutation test.

**Results:**

The proposed permutation test leads to smaller *p*-values in comparison to the original two-part test. We found this for both DNA methylation data and microarray data. With a simulation study we confirmed this result and could show that the two-part permutation test is, on average, more powerful. The new test also reduces, without any loss of power, to a standard test when there are no null or truncated values.

**Conclusion:**

The two-part permutation test can be used in routine analyses since it reduces to a standard test when there are positive values only. Further advantages of the new test are that it opens the possibility to use other test statistics to construct the two-part test and that it avoids the use of any asymptotic distribution. The latter advantage is particularly important for the analysis of microarrays since sample sizes are usually small.

## Background

The addition of a methyl group at the carbon-5 position of cytosine is a modification of DNA called DNA methylation. In mammalian cells, DNA methylation is essential for proper development [1]. The methylation patterns of tumor cells are altered compared to those of normal cells, moreover, there are also differences between different types of cancer as shown for subtypes of leukemia [2] and lung cancer [3]. Thus, DNA methylation analysis promises to become a powerful tool in cancer diagnosis [4].

DNA methylation data can be obtained using the MethyLight technology [5]. When the tested region is not or only partially methylated the result is negative (undetectable methylation, null values). In contrast, samples that show methylation will have a value greater than 0 [4]. Thus, DNA methylation data obtained with MethyLight have a clump of zero observations and a continuous nonzero part. For such a data structure, two-part models as proposed by Lachenbruch [6-8] are applicable. In that approach, the test statistic is the sum of two squared statistics, one comparing the proportions of zeros and one comparing the positive values. For example, one can use the binomial test and the Wilcoxon rank sum test. The asymptotic null distribution of the sum of the squares of the two test statistics is χ^2 ^with two degrees of freedom (df = 2).

In microarray data it is relatively common that small and negative expression levels were clipped-off to be equal to an arbitrarily chosen cutoff value. Recent examples used different cutoff values: 1, 20, and 50, respectively [9-11]. Aside from the fact that negative values make no biological sense, there are, with regard to oligonucleotide arrays from Affymetrix, two primary reasons for truncating the values [12]. First, spots at the low intensity range are generally more vulnerable to noise, thus, it is thought that the technology produces a poor discrimination at low levels of expression [13]. Second, the focus is on expression of identified genes and expressed sequence tags. Differences at negative or low values may result from differences in binding to the mismatch probes. Since it is generally not known what binds to the mismatch probes, the differences at negative or low values cannot be attributed to target genes.

Due to the truncation there are two different types of data: truncated values and original observations. Since the truncated values are not just another point on the continuum of possible values, it would be inappropriate to use a standard statistical method that would treat all values equally [14]. The two different types of data should be analyzed separately. Therefore, the two-part model, comparing the proportions of truncated values and the distribution of positive values, is applicable. Note that negative expression levels are not possible when the Affymetrix Microarray Suite (MAS) 5.0 software is used. However, small values are possible and may be truncated.

Since, in microarray experiments, the sample sizes, i.e. the numbers of replications, are usually very small [15,16], the use of the asymptotic distribution of Lachenbruch's two-part test statistic may be questionable. Thus, we carried out permutation tests with the two-part statistic. For a permutation test all possible permutations under the null hypothesis are generated. In our situation we permute the group labels for the whole sample, i.e. for truncated values (or the null values in case of methylation data) and original observations. Then, the test statistic is calculated for each permutation. The null hypothesis can then be accepted or rejected using the permutation distribution of the test statistic, the *p*-value being the probability of the permutations giving a value of the test statistic as supportive or more supportive of the alternative than the observed value [17,18]. Thus, inference is based upon how extreme the observed test statistic is relative to other values that could have been obtained under the null hypothesis.

We found that, in the case of a two-part model, the permutation test is not only a way to avoid the use of an asymptotic distribution, but also is a more powerful test, i.e. a test that produces, on average, smaller *p*-values. In addition, the permutation test reduces, without any loss of power, to a single test if no truncated (or null) values were present. Thus, the proposed test is applicable in routine use whether or not truncated (or null) values occur. After the definition of the tests in the following section, we present our findings for DNA methylation data and microarray data. We then confirm the results using simulations.

### Two-part tests

As briefly mentioned above a two-part test statistic is the sum of two squared statistics, one comparing the proportions of truncated values and one comparing the positive values. Let *n*_1 _and *n*_2 _be the numbers of independent observations regarding one gene (or one region in case of methylation data, respectively), for two groups to be compared. The observed numbers of truncated values (or null values in case of methylation data) in the two groups are denoted by *m*_1 _and *m*_2_. To compare these numbers *m*_1 _and *m*_2 _Lachenbruch [6] used the statistic


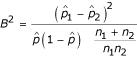


where 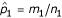, 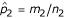, and 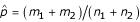. Under the null hypothesis the proportions of truncated values are not different between the two groups, and *B*^2 ^is asymptotically χ^2^-distributed with df = 1. *B*^2 ^is always well defined, unless there are only truncated values in both groups or no truncated values at all. For these two extreme cases we set *B*^2 ^= 0.

For the second part in Lachenbruch's two-part model one can use different tests, Lachenbruch [6-8] considered the Wilcoxon rank sum test, Student's *t *test, and the Kolmogorov-Smirnov test. The use of the latter test in a two-part model, however, was too liberal, the type I error rate was close to 0.065 (for a significance level of α = 0.05 [7,8]). Both other tests work well. Here, we apply the Wilcoxon test for two reasons. This test was used in the original analyses of the data we use below [3,11]. Nonparametric tests based on ranks are more appropriate for non-normally distributed data such as microarray data [19].

The standardized rank sum statistic based on the non-truncated (or positive values in case of methylation data) values is defined as


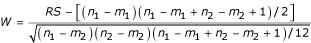


where *RS *is the rank sum, i.e. the sum of the ranks in group 1. When there are ties within the non-truncated values the denominator slightly changes [[20], p. 109]. For the extreme case that there are only truncated values in at least one group we set *W *= 0.

The test statistic for the two-part test is *X*^2 ^= *B*^2 ^+ *W*^2^. Under the null hypothesis of no difference between the groups, *X*^2 ^is asymptotically χ^2^-distributed with df = 2 [6,7]. Alternatively, a permutation test can be performed with the statistic *X*^2^. This test, called two-part permutation test here, is a permutation test based on the sum statistic *X*^2^. It is carried out by permuting the group labels for the whole sample. Thus, all observations, truncated and non-truncated values (or null and positive values in case of methylation data) are reallocated to the groups. When performing this two-part permutation test the exact permutation distribution of *X*^2 ^is determined. This distribution, computed by generating all possible permutations or, for the *p*-values given below in Table 1, using a simple random sample of 20,000 permutations, is used to compute the *p*-value. Since it is a permutation test based on the sum *X*^2^, it is neither necessary to determine the permutation distributions of the summands *B*^2 ^and *W*^2 ^nor to calculate the *p*-values of the univariate tests related to *B*^2 ^and *W*^2^.

### Application to actual methylation data

We use DNA methylation data from 7 regions and 87 lung cancer cell lines, 41 lines are from small cell lung cancer and 46 lines from non-small cell lung cancer [3,4]. The proportion of positive values for the different regions ranges from 39 to 100% for the small cell lung cancer and from 65 to 98% for the non-small cell lung cancer. The data are available at . Siegmund et al. [4] transformed the data by standardizing the positive values on the natural-log scale. However, for the tests applied here, this transformation has no influence.

Table 1 presents the *p*-values of the two tests. For most regions the two-part permutation test gives a smaller *p*-value than the original two-part test. The only exception is the region *APC*. However, the original two-part test's *p*-value for this region is 0.1684 and, for a *p*-value of this size, a small change in the value is usually of no importance.

### Application to actual microarray data

**Figure 1:**
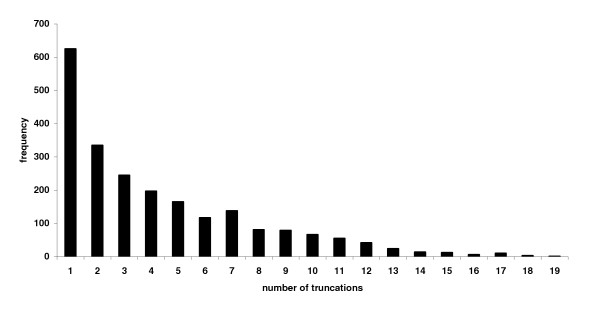
Number of genes with the given number of truncations per gene (data from the microarray experiment of Tschentscher et al. [11], only genes with at least one truncation)

Tschentscher et al. [11] performed an experiment with HG-U95Av2 oligonucleotide arrays in order to compare patients with uveal melanomas with and without monosomy 3. Expression values were calculated by use of the MAS 4.0 software. The data are available at . The sample size in this microarray experiment is 10 per group. As mentioned above, expression levels below 50 were set to 50. This data truncation occurred in 2,215 (28%) out of 7,902 genes. First, we consider these 2,215 genes. Figure 1 displays the number of genes for the different number of truncated values per gene.

Table 2 shows the frequencies of different size groups of the *p*-values. Often, the two-part permutation test gives a smaller *p*-value than the original two-part test. For instance, for 19 genes the *p*-value is ≤ 0.001 when the latter test is applied. The permutation test gives a *p*-value ≤ 0.001 for these 19 and for 39 additional genes. As usual, the majority of genes do not show any indication of being differentially expressed. For these genes a slight change in the *p*-value is of no importance. Thus, in Table 3, we consider the genes for which the *p*-value of the original two-part test is ≤ 0.1. Out of the 2,215 genes 514 remain. As shown in Table 3 the *p*-values of the two-part permutation test are, on average, distinctly smaller than those of the original two-part test.

For a large proportion of genes (72% in this data set) there are no truncated values. In that case, the two-part statistic reduces to the sum 0 plus the squared standardized rank sum *W*^2^. Of course, one has to define a priori whether the two-part test or the Wilcoxon test will be used to analyze the data. If the original two-sided (asymptotic) Wilcoxon test were chosen and applied, one could compare *W*^2 ^with critical values from a χ^2 ^distribution with one degree of freedom (df). If the two-part were chosen there is df = 2 and, in case of no truncated values, power is lost compared to the original Wilcoxon test. For instance, the 95% percentile of the χ^2 ^distribution with df = 1 is 3.84, but it is 5.99 for df = 2. The permutation test using the sum statistic *X*^2 ^does not suffer from this power loss: When there are no truncated values there is, of course, no difference in the proportions of truncated values, and the test statistic *X*^2 ^is, for every permutation, the sum 0 + *W*^2^. Thus, the two-part permutation test reduces to the exact two-sided Wilcoxon rank sum test when there are no truncated values. Consequently, this permutation test does not only give smaller *p*-values, but it is also applicable in routine use whether or not truncated values are present.

### Simulation study

The two different tests, the original two-part test and the two-part permutation test, were compared in a Monte Carlo simulation study performed using SAS version 8.2, 5,000 simulation runs were generated for each configuration. The sample size of 10 per group was chosen as in the microarray experiment presented above. In some configurations some randomly chosen values were set to 0 according to binomial distributions with the probabilities *p*_1 _and *p*_2_, and the remaining observations were generated according to a lognormal distribution (with median 1 and σ = 1). Then, the values of one group were shifted if applicable. In some other configurations all observations were generated according to the lognormal distribution and, in one group, shifted. Then, values smaller than a cutoff value were truncated.

The type I error rates of the two tests are very similar. With e.g. *p*_1 _= *p*_2 _= 0.3 and a significance level of α = 0.05 the simulated type I error rates were 0.049 for both the original two-part test and the two-part permutation test.

Table 4 displays results for situations with a difference between the two groups. As above, only those comparisons were regarded for which there is some indication of a difference, i.e. a *p*-value ≤ 0.1 of the original two-part test. In all considered configurations the median of the difference between the *p*-values of the original two-part test and the two-part permutation test is positive. The finding that the *p*-values of the permutation test are smaller corresponds to a higher power of this test. The power is given in Table 5, as shown the power of the two-part permutation test is at least as high as that of the original two-part test. There is only one exception, the latter test is slightly more powerful in one situation, i.e. when the proportion of zeros is higher in group 1 and the positive values are larger in group 1.

## Discussion

Previous research demonstrated that a two-part test is appropriate and powerful in the presence of a clump of zero observations (i.e. truncated or null values). In this paper we propose a permutation test for such situations with two types of data. Usually, nonparametric tests can be performed based on an asymptotic distribution or based on a permutation null distribution. The two approaches often give similar results, especially when the sample sizes are large. However, in the case of a two-part test one cannot simply replace the asymptotic distributions of *B*^2 ^and *W*^2 ^by the exact permutation distributions. If so, one would compute two exact *p*-values although the aim of a two-part test is to receive one *p*-value that combines information from both parts. Therefore, the two-part permutation test uses the exact permutation distribution of the sum statistic *X*^2^. That this permutation distribution of the sum is generated rather than to simply replace the asymptotic distributions of the summands by their exact permutation distributions may be the reason why the permutation test is more powerful.

A disadvantage of a permutation test is that it can be computer-intensive. However, this issue is less relevant now due to faster algorithms [[18], chap. 13] and the advent of high-speed PCs. Furthermore, one can carry out a permutation test based on a random sample out of the possible permutations, as we did for the DNA methylation data (see Table 1).

In microarray experiments it is common to investigate thousands of genes simultaneously. The approach presented here for the identification of differentially expressed genes is to consider a univariate testing problem for each gene. A correction for the multiplicity of genes is a subsequent step, that is, like the previous step of normalizing the data, outside the scope of this paper. A common approach to the multiplicity problem is to consider a procedure for testing the genes simultaneously for differential expression with the test on an individual gene being implied in the simultaneous test. For such a procedure different proposals have been made recently. For instance, there are methods based on the *p*-values of the tests from individual genes [21-23]. In a similar manner, the multiplicity of regions can be managed in DNA methylation data.

## Conclusion

Aside from the shown improvement in power, the proposed two-part permutation test has three important advantages. First, it avoids the use of any asymptotic distribution and, therefore, can safely be applied in case of small sample sizes that are common in microarray experiments. Second, it reduces without any loss of power to the exact Wilcoxon test if there were no truncated (or zero) values. Thus, it can be used in routine analyses. Third, the permutation test opens the possibility to use other tests to construct the two-part test. Thus, tests with unknown or non-standard null distributions can be used. For instance, one could replace the Wilcoxon test by the Baumgartner-Weiß-Schindler test [24] that was recently recommended for the analysis of gene expression data [19].

## Authors' contributions

MN performed the statistical analyses and drafted the manuscript. TB prepared the microarray data. TB and KHJ participated in the design of the simulation study and helped to draft the manuscript. All authors read and approved the final manuscript.

## Figures and Tables

**Table 1:** *p*-values of the original two-part test and the two-part permutation test for seven regions in lung cancer cell lines; DNA methylation data from Siegmund et al. [4]

Region	Original two-part test	Two-part permutation test^1^
*CALCA*	*p *= 0.0005	*p *= 0.0004
*PTGS2*	*p *= 0.0002	*p *= 0.0001
*MTHFR*	*p *= 0.0007	*p *= 0.0002
*MGMT-M1*	*p *= 0.0860	*p *= 0.0857
*APC*	*p *= 0.1684	*p *= 0.1712
*MYOD1*	*p *= 0.0132	*p *= 0.0111
*ESR1*	*p *= 0.0040	*p *= 0.0030

^1^performed based on simple random samples of 20,000 permutations (when using 20,000 permutations a 95%-confidence interval for the p-value is the observed p-value ± 0.007 when *p *= 0.5, or ± 0.003 when *p *= 0.05).

**Table 2:** Frequencies of different size groups of the *p*-values of the original two-part test and the two-part permutation test for genes with at least one truncation; data from the microarray experiment of Tschentscher et al. [11]

*p*-value of the original two-part test	*p*-value of the two-part permutation test				
	≤ 0.001	> 0.001 and ≤ 0.01	> 0.01 and ≤ 0.05	> 0.05 and ≤ 0.1	> 0.1
≤ 0.001	19	0	0	0	0
> 0.001 and ≤ 0.01	39	56	0	0	0
> 0.01 and ≤ 0.05	0	50	164	8	0
> 0.05 and ≤ 0.1	0	0	49	112	17
> 0.1	0	0	0	53	1648

**Table 3:** Differences between the *p*-values of the original two-part test and the two-part permutation test for genes with at least one truncation and small *p*-values (i.e. the *p*-value of the original test must be as small as mentioned under condition), a positive difference means that the two-part permutation test has a smaller p-value than the original test; data from the microarray experiment of Tschentscher et al. [11]

Condition	Number of remaining genes	Mean difference (± SD)	Median difference	Quartiles of the difference
*p *≤ 0.1	514	0.0065 (± 0.0126)	0.0047	0.0012, 0.0124
*p *≤ 0.05	336	0.0057 (± 0.0064)	0.0040	0.0018, 0.0095
*p *≤ 0.01	114	0.0025 (± 0.0018)	0.0019	0.0010, 0.0035
*p *≤ 0.001	19	0.0006 (± 0.0003)	0.0006	0.0003, 0.0009

**Table 4:** Results of the simulation study: Differences between the *p*-values of the original two-part test and the two-part permutation test for data sets with small *p*-values (i.e. *p*-value of the original test ≤ 0.1), a positive difference means that the two-part permutation test has a smaller *p*-value than the original test; *p*_1 _and *p*_2 _are the probabilities for zero values and the positive values in group 1 are shifted by μ; 5,000 data sets with *n*_1 _= *n*_2 _= 10 were generated for each configuration

Configuration	Number of data sets	Mean difference (± SD)	Median difference	Quartiles of the difference
*p*_1 _= *p*_2 _= 0, μ = 2.5	4538	0.0118 (± 0.0145)	0.0057	0.0013, 0.0161
*p*_1 _= *p*_2 _= 0.3, μ = 2.5	4038	0.0008 (± 0.0069)	0.0020	0.0008, 0.0033
*p*_1 _= 0.4, *p*_2 _= 0.2, μ = 2.5	4346	0.0010 (± 0.0059)	0.0019	0.0008, 0.0033
*p*_1 _= 0.2, *p*_2 _= 0.4, μ = 2.5	4270	0.0011 (± 0.0056)	0.0018	0.0006, 0.0030
*p*_1 _= 0, *p*_2 _= 0.3, μ = 2.5	4883	0.0039 (± 0.0055)	0.0020	0.0005, 0.0051
*p*_1 _= 0.3, *p*_2 _= 0, μ = 2.5	4893	0.0040 (± 0.0053)	0.0022	0.0007, 0.0049
*p*_1 _= 0, *p*_2 _= 0.4, μ = 0	3427	-0.0033 (± 0.0119)	0.0006	-0.0092, 0.0029
cutoff value = 0.5, μ = 2.5	4621	0.0059 (± 0.0081)	0.0034	0.0009, 0.0072
cutoff value = 1, μ = 2.5	4860	0.0017 (± 0.0052)	0.0013	0.0003, 0.0032

**Table 5:** Results of the simulation study: Powers of the original two-part test and the two-part permutation test; 5,000 data sets with *n*_1 _= *n*_2 _= 10 were generated for each configuration (notation as in Table 4, significance level α = 0.05)

Configuration	Original two-part test	Two-part permutation test
*p*_1 _= *p*_2 _= 0, μ = 2.5	0.82	0.93
*p*_1 _= *p*_2 _= 0.3, μ = 2.5	0.67	0.67
*p*_1 _= 0.4, *p*_2 _= 0.2, μ = 2.5	0.76	0.75
*p*_1 _= 0.2, *p*_2 _= 0.4, μ = 2.5	0.74	0.74
*p*_1 _= 0, *p*_2 _= 0.3, μ = 2.5	0.93	0.96
*p*_1 _= 0.3, *p*_2 _= 0, μ = 2.5	0.95	0.97
*p*_1 _= 0, *p*_2 _= 0.4, μ = 0	0.45	0.47
cutoff value = 0.5, μ = 2.5	0.85	0.90
cutoff value = 1, μ = 2.5	0.92	0.92
